# Is There a Role for Neoadjuvant Targeted Therapy to Downsize Primary Tumors for Organ Sparing Strategies in Renal Cell Carcinoma?

**DOI:** 10.1155/2012/250479

**Published:** 2012-06-20

**Authors:** A. Bex, B. K. Kroon, R. de Bruijn

**Affiliations:** Division of Surgical Oncology, Department of Urology, The Netherlands Cancer Institute, Plesmanlaan 121, 1066 CX Amsterdam, The Netherlands

## Abstract

With an increasing number of small renal masses being diagnosed organ-preserving treatment strategies such as nephron-sparing surgery (NSS) or radiofrequency and cryoablation are gaining importance. There is evidence that preserving renal function reduces the risk of death of any cause, cardiovascular events, and hospitalization. Some patients have unfavourable tumor locations or large tumors unsuitable for NSS or ablation which is a clinical problem especially in those with imperative indications to preserve renal function. These patients may benefit from downsizing primary tumors by targeted therapy. This paper provides an overview of the current evidence, safety, controversies, and ongoing trials.

## 1. Introduction 

Advances in imaging have increased the number of incidentally diagnosed small renal masses (SRMs) [1]. As a consequence, surgical management of these small tumors is shifting from radical nephrectomy to nephron-sparing surgery (NSS) [2]. The development of minimally invasive techniques has introduced alternatives in the form of laparoscopic (robotic) partial nephrectomies and ablative techniques such as radiofrequency ablation (RFA) or cryoablation (CA) [3–5]. The rationale for preserving nephrons is based on recent evidence from large epidemiological studies that clearly demonstrate that preservation of renal function reduces the risk of death of any cause, cardiovascular events, and hospitalization.

In a large study the adjusted hazard ratio for death from any cause, cardiovascular events, and hospitalization among 1,120,295 ambulatory adults, according to the estimated GFR, was investigated. In the range of 15–29 mL/min/1.73 m^2^ the adjusted hazard ratio was 3.2 [6] while it was 1.0 in the range >60 mL/min/1.73 m^2^. In a large epidemiological retrospective cohort study based on linked Surveillance, Epidemiology, and End Results-Medicare (SEER) data the authors identified 10,886 patients who underwent partial or radical nephrectomy. Among contemporary patients, partial nephrectomy was associated with less clinically apparent renal morbidity than radical nephrectomy. Patients who underwent partial nephrectomy experienced fewer adverse renal outcomes (16.4% versus 21.8%; adjusted hazard ratio, 0.74; 95% confidence interval (CI), 0.58–0.94), including a trend toward less frequent receipt of dialysis services, dialysis access surgery, or renal transplantation [7]. Another group identified from the SEER registry 4,216 patients with histologically confirmed renal cell carcinoma 2 cm or less who were treated with partial or radical nephrectomy [8]. Radical nephrectomy was associated with worse overall and cardiovascular survival compared to partial nephrectomy in patients with localized renal cell carcinoma 2 cm or less. To minimise a potential confounding effect of cancer mortality on the interpretation of these epidemiological data, one study compared overall survival (OS) in a subset of patients with unanticipated benign SRM [9]. On retrospective multivariate cohort analysis, controlling for both comorbidity and age, radical nephrectomy was associated with a 2.5-fold increased risk of death compared to partial nephrectomy (95% CI, 1.3–5.1).

In a systematic review including 69 articles for final evaluation it was concluded that partial nephrectomy for cortical tumours of ≤4 cm has higher morbidity but similar oncological outcome as total nephrectomy with a reduction of loss of renal function and a potential survival benefit regardless of tumor stage [10]. Recently OS data of the only randomized trial performed comparing partial nephrectomy to radical nephrectomy were reported [11]. Though in the intention-to-treat analysis partial nephrectomy was significantly less effective than radical nephrectomy in terms of OS, this statistical significant difference was not reproduced when the analysis was performed in the target population of clinically and pathologically eligible RCC patients. Only 12 of 117 deaths in the entire study were renal cancer related. Recent guidelines and reviews therefore recommend that partial nephrectomy should be standard for cortical tumours ≤4 cm [10, 12] which may be extended to tumors of up to 7 cm. Some authors argue, that it is not the primary tumor size that limits the approach, but the location.

Some patients have a small renal mass with an unfavourable location within the kidney. The PADUA and RENAL scores have been recently developed and validated to allow a standardized measurement of this complexity and to allow comparison of data in future trials [13]. The scores are simple and based on imaging criteria. Based on a scale of points the PADUA score [14] allows to classify patients into low (6-7), moderate (8-9), and high (>/=10) complexity tumors. Equally, the RENAL score categorizes into low (4–6), moderate (7–9), and high (10–12) complexity tumors [15]. Especially patients with a moderate-to-high complexity have a higher risk of surgical morbidity and of conversion to total nephrectomy. These patients may therefore benefit from tumor downsizing by targeted therapies before approaching the tumor by NSS or ablative techniques. In addition, size is often the limiting factor for ablative techniques. More central tumors and those exceeding 4 cm in size have a higher complication rate and worse outcome [16]. In addition, patients with an imperative indication for nephron-sparing surgery but locally extensive tumors otherwise requiring nephrectomy pose a clinical problem [17].

Downsizing renal tumors for organ-preserving strategies may therefore be an attractive concept, as it may allow more patients to benefit from preserved renal function who would otherwise be candidates for nephrectomy. After the advent of targeted therapy responses were seen for the first time in primary tumors of patients with metastatic RCC, which suggested that downsizing by pretreatment could be used as a treatment modality. Meanwhile studies and series report on the neoadjuvant use of these drugs in the nonmetastatic setting. This review aims to define the current role of neoadjuvant targeted therapy to downsize localized RCC to facilitate nephron-sparing strategies. 

## 2. Methods

A literature search was performed using MEDLINE and Pubmed, and publications on SRM, NSS, ablative techniques, partial and radical nephrectomy, neoadjuvant and presurgical therapy with receptor tyrosine kinase inhibitors (TKIs), vascular endothelial growth factor (VEGF) antibodies, mammalian target of rapamycin inhibitors (mTORs) relating to nonmetastatic and metastatic RCC were retrieved and critically reviewed. In addition, registries of clinical trials (e.g., http://www.clinicaltrials.gov/) were searched. Abstracts of meeting proceedings were included in the paper, to ensure a timely overview of a rapidly evolving field.

## 3. Downsizing with Targeted Therapies

The introduction of novel drugs that target angiogenesis has resulted in an improvement in treatment of mRCC. Currently approved agents include receptor TKIs, VEGF antibodies, and mTOR inhibitors [18]. 

In patients with mRCC, targeted therapy results in progression-free survival (PFS) of up to 15 months and an overall survival (OS) of 26 months, which may even reach 4 years with sequential therapy [19, 20].

Targeted therapy has been shown to have limited effect on the primary tumor [21]. Most of the evidence stems from retrospective studies and smaller phase II trials of patients with metastatic RCC. Though 85% of all patients reveal a reduction in longest diameter, the median downsizing is approximately 10%, and only 5% have a true partial remission of the primary lesion by RECIST criteria. Therefore neoadjuvant treatment of nonmetastatic RCC to downsize tumors is regarded as controversial. However, several phase II studies have revealed that the response at metastatic sites is more prominent than in the primary tumour [22, 23]. Though this may be in part due to a different biology between primary tumour and metastatic size, another potential argument may be that a better size reduction is correlated to a smaller initial tumor size. A recent publication suggests that this may indeed be the case [24]. It may be that smaller primary tumors develop a more prominent downsizing in a higher percentage than may seem from the previous publications. Often, large primary tumours of metastatic patients have been included in previous studies and retrospective series [21–23, 25]. However, data on smaller tumors have been published as part of larger studies and patient series [26–29]. Especially patients with primary tumors between 5 and 7 cm may benefit from further downsizing as they may become candidates for ablative techniques or pretreatment may facilitate nephron-sparing surgery in complex cases. One retrospective study looked specifically into neoadjuvant therapy and subsequent nephron sparing surgery [28]. Twelve patients received sunitinib before NSS for imperative indications. The mean pretreatment diameter of the primary tumor was 7.1 cm which was reduced by a mean diameter of 1.5 cm (21.1%). Four of 14 primary tumors had a partial response. Eventually NSS was performed in all cases, including those 10 tumors with stable disease. The mean warm ischemia time was 22.5 minutes. Long-term outcomes were comparable to nonpretreated NSS. In addition, case reports observed an improvement of the RENAL nephrometry score after pretreatment. Gorin et al. describe a dramatic downsizing of bilateral tumors in a patient receiving neoadjuvant sunitinib 50 mg/day for four cycles interrupted 3 weeks before NSS [30]. The RENAL score improved by 1-2 points which is attributed to a change in size. Ansari et al. reported on a similar case with bilateral tumors treated successfully by NSS after neoadjuvant sunitinib [17]. In order to estimate the probability of downsizing in correlation to primary tumor size we conducted a literature search and pooled our own data with larger retrospective series and prospective trials in which patients were treated with the tyrosine kinase inhibitors sunitinib and sorafenib and in which pre- and posttreatment tumor sizes were individually reported (Kroon B., primary tumor downsizing in renal cell carcinoma is more prominent in smaller tumors enabling nephron sparing strategies, presented at the 27th Annual EAU Congress, Paris, 2012). 

Only few studies report on individual pre- and posttreatment tumor sizes. Collectively, 89 primary tumors ranging from <5 cm to >10 cm, most of them from primary metastatic RCC patients, could be evaluated. Pre- and posttreatment sizes were calculated according to RECIST. Smaller tumor size was related to more effective downsizing. In tumors <5 cm median downsizing was 32% (−46 to +11%) and in those with tumors between 5 and 7 cm 11% (−55 to +16%). However, 38% of those with tumors between 5 and 7 cm reduced into a range of 2.3–4.7 cm in which ablative techniques are principally feasible and organ-preserving strategies may benefit from reduced size. In tumors >7 cm the likelihood to downsize tumors is extremely low. This observation suggests that the smaller the tumor, the greater the likelihood and the more effective the downsizing. However, there is still a majority of patients who do not experience downsizing of the primary tumor despite a smaller initial tumor size. Without predictive clinical or biomarkers neoadjuvant targeted therapy outside trials should therefore be reserved for those few patients with an imperative indication for preserving renal function who would otherwise have to undergo nephrectomy and dialysis. Patients should be counseled, and a decision should be based on informed consent after having discussed the potential advantages and disadvantages (Table 1). Most of the present evidence has been gathered with the tyrosine kinase inhibitor sunitinib, including one study with sorafenib [21–23, 25–29].

M-TOR Inhibitors are considered unsuitable for downsizing strategies as they often lead to stabilization of disease rather than reduction of volume or longest diameter [31]. Monoclonal antibodies against circulating VEGF such as bevacizumab have the disadvantage of a long half-life limiting their use in combination with surgery [32]. Therefore, the current choice of drugs is limited, and further prospective studies including the recent more potent TKI are warranted. 

## 4. Prospective Studies in Nonmetastatic RCC

Presently, only two prospective phase II studies have been completed and published in which patients with nonmetastatic RCC were treated with neoadjuvant sunitinib or sorafenib [26, 27]. In both trials, primary metastatic patients were included, and the primary endpoint was efficacy and safety, rather than the ability to downsize tumors for organ-sparing strategies. The sunitinib trial was restricted to patients with clear-cell RCC, and 85% of patients had some degree of tumor shrinkage [27] which was higher than the downsizing achieved with sorafenib. In the sunitinib trial 67% of the patients were nonmetastatic, and patients were treated with a continuous dose of 37.5 mg/day for 3 months. The median tumor shrinkage was −11.8% (range −27–+11%). In 40% of the patients a partial nephrectomy was performed. The lower dose of sunitinib instead of the standard 50 mg/day may be responsible for a limited effect on downsizing. Metastatic lesions showed a lesser RECIST response in patients receiving continuous sunitinib at 37.5 mg/day when compared to 50 mg/day (4/2 regimen) [33]. In addition, it is unclear if NSS had not been an option before neoadjuvant therapy leaving the question whether neoadjuvant treatment facilitated organ preservation unanswered. This question is currently addressed in a phase II study examining the efficacy of pazopanib when administered prior to surgery [34]. The trial enrolls patients with impaired renal function and large tumours requiring otherwise a radical nephrectomy with the aim to downsize and perform NSS (NCT01158521). Primary endpoint is rate of conversion from nephrectomy to NSS. Additional trials are recruiting which include patients with non-metastatic RCC for neoadjuvant treatment with pazopanib and axitinib, though they do not specifically address downsizing for organ-sparing surgery [35, 36]. This neoadjuvant pazopanib study includes patients with non-metastatic RCC T2 and greater who will receive 800 mg orally once daily for 12 weeks [35]. Primary endpoint is RECIST response rate. In the axitinib trial patients with locally advanced but non-metastatic RCC are included to receive 5 mg by mouth twice each day for 12 weeks [36]. The primary endpoint is response in the primary tumor. However, both trials will reveal percentage of downsizing which is expected to be prominent with axitinib, a more potent TKI of the latest generation. In addition, both trials have the potential to establish a predictive biomarker profile associated with substantial downsizing which may ultimately help to select future patients for this approach.

## 5. Safety of Neoadjuvant Therapy

Since patients with localized RCC can principally be cured by surgery alone, any potential neoadjuvant treatment requires to be surgically and oncologically safe. Of concern are not only surgical complications that may be associated with targeted therapy but further tumor growth and metastasis in the pretreatment period. 

The surgical safety of targeted therapy has been extensively reported, and with the exception of bevacizumab with its long half-time of 3 weeks, TKIs such as sunitinib or sorafenib have been interrupted as close as 24 hours before surgery without serious adverse events [32, 37]. There is a 9–11% incidence of superficial wound healing impairments of Clavien grade I reported for both drugs after cytoreductive surgery. As these figures are from nonrandomized studies, it is currently unknown if this percentage is higher than after upfront nephrectomy. In addition, cytoreductive nephrectomy is often performed in larger tumors requiring extensive surgery, and the incidence may be lower for limited interventions with smaller masses. Laparoscopic total and partial nephrectomies have been performed subsequent to sunitinib without wound healing problems [23]. Surgeons evaluated dissection of tissue and performing total and partial nephrectomies differently, and descriptions are subjective. Some observed difficulties due to fibrosis whereas others experienced dissection facilitated by edema in the tissue planes [23]. 

Neoadjuvant therapy results in a delay of curative surgery by at least 2 to 3 months as most of the downsizing occurs in the first months [38, 39]. This may be potentially harmful as tumor progression may occur. In retrospective series on 168 primary metastatic tumors an increase by >30% has been observed in 1.19% and increases >11% in 4.76% [21]. Individual cases have been reported of new onset caval vein thrombi leading to more extensive surgery [40]. Of even bigger concern may be the onset of metastasis in the pretreatment period. Translational data from a seminal preclinical mouse model suggest that a short treatment with sunitinib increases metastasis [41]. A potential explanation may be the reduction of pericytes with ensuing hypoxia that drives mesenchymal-endothelial transformation and an increase in tumor stem cells, which has been observed in two recent mouse models [42, 43]. It has often been criticized that animal models are fundamentally flawed and that increased metastasis is not observed in clinical practice after treatment interruption. However, interruption of treatment for metastatic patients is often short, as in the 2-week interval of sunitinib treatment, and the reintroduction of therapy may mask this effect. This may be a different risk for neoadjuvant therapy where therapy is discontinued following surgery. Currently, there is no clinical evidence for rapid disease progression from neoadjuvant phase II trials in non-metastatic disease. In primary metastatic disease, however, rapid disease progression in the 4-week treatment break following surgery has been observed in up to 30% of patients following pretreatment with sunitinib [44]. Most of these metastatic patients stabilize after reintroduction of treatment, and it is currently unknown whether this progression in the treatment break adversely affects outcome. For patients with non-metastatic local tumors, however, who can be cured by surgery alone, the potential of antiangiogenic agents to drive tumors into a hypoxic and metastatogenic state needs to be clarified before initiation of large phase III trials. 

## 6. Summary and Conclusion

An ideal neoadjuvant treatment to downsize renal tumors to facilitate surgical or ablative approaches would combine a high probability of success in a large percentage of patients with little adverse effects. The current results from reports on downsizing primary tumors in the non-metastatic settings suggest that the smaller the tumor, the greater the likelihood and the more effective the downsizing. A potential benefit of neoadjuvant treatment with sunitinib or sorafenib to downsize the primary tumor for ablative techniques or NSS may exist in the group 5–7 cm size. Small tumors of <5 cm seem to have the most prominent downsizing. However, this is unlikely to be of clinical benefit, unless NSS is expected to be complex and difficult according to the RENAL or PADUA score. Nephrometry scores are an attractive tool to estimate the complexity of partial nephrectomy [13], and an improvement of the score following neoadjuvant therapy may suggest an easier procedure. Contrarily, it is very likely that neoadjuvant therapy has an effect on tumor size only and will not change the anatomical position of the renal mass. Therefore, centrally or unfavourably located tumors may remain difficult to access for both partial nephrectomy or ablative techniques after downsizing [10, 16]. With the exception of some case reports there are no results from prospective trials that report specifically if partial nephrectomy benefited from neoadjuvant treatment [17, 30]. This leaves the fundamental question unanswered whether downsizing of a renal mass is improving partial nephrectomies or whether this remains essentially a surgical problem depending on competence and skill. There are recent data that suggest that a successful partial nephrectomy is not depending on the size but the location of a renal tumor [45]. 

Due to the absence of predictive biomarkers, the percentage of substantial downsizing is still too unpredictable to recommend this approach outside clinical trials. In addition, with the exception of those patients who have a (functional) single kidney bearing an extensive tumor unsuitable for organ-preserving strategies, the majority of patients with large primary non-metastatic tumors unsuited for organ-preserving and a normal contralateral kidney do not necessarily need to undergo NSS or ablation after downsizing. Delaying the established paradigm of curative nephrectomy with the risk of disease progression under neoadjuvant treatment is of concern, which is supported by data from preclinical models. In addition, recent data suggest that smaller masses are often undergraded at biopsy [46]. At present there are only few reports suggesting that successful NSS has been achieved because of neoadjuvant downsizing. In a recent survey among members of the Society of Urologic Oncology (SUO), 98% of respondents support evaluating the use of pretreatment targeted agents for high-risk locally advanced and metastatic RCC, whereas a lower number (70%) was supportive of neoadjuvant therapy for localized disease [47]. This reflects a prevailing scepticism to downsize tumors that may be cured by surgery alone. However, there is a clinical need to further evaluate the potential to downsize tumors predictably for those patients who have an imperative indication to preserve renal function. Several phase II studies with potentially more effective targeted agents are ongoing and may reveal predictable downsizing and biomarker profiles in patients with primary renal tumors otherwise requiring radical nephrectomy.

## Figures and Tables

**Table 1:** 

Pro neoadjuvant therapy	Contra neoadjuvant therapy
(i) Downsizing may allow organ preservation and preservation of renal function in patients with impaired renal function otherwise requiring nephrectomy and dialysis.	(i) The percentage of patients experiencing substantial downsizing with sunitinib or sorafenib is unpredictable.
(ii) Nephron sparing is associated with improved overall survival and reduced morbidity. Downsizing tumors for nephron sparing strategies in patients otherwise requiring nephrectomy may improve long-term survival.	(ii) There are currently no biomarkers predicting local tumor response.
(iii) Case reports and retrospective series suggest that patients with tumors of 5–7 cm in size may have a benefit of downsizing tumors with sunitinib and sorafenib followed by NSS or ablation.	(iii) Primary tumors that can be cured by surgery alone may progress under neoadjuvant therapy.
(iv) The chance to have a marked downsizing in primary tumors of 5–7 cm is substantially higher than for larger tumors.	(iv) Preclinical models suggest an increased metastatic potential of solid tumors after targeted therapy.
	(v) Neoadjuvant therapy may be associated with wound healing impairments.
	(vi) Drug-related adverse events may further delay curative surgery.
